# Coronary artery perforation secondary to lifesaving pericardiocentesis for cardiac tamponade: a case report

**DOI:** 10.1186/s12872-021-01875-0

**Published:** 2021-01-28

**Authors:** Daisuke Kanda, Takuro Takumi, Takeshi Sonoda, Ryo Arikawa, Kazuhiro Anzaki, Yuichi Sasaki, Mitsuru Ohishi

**Affiliations:** Department of Cardiovascular Medicine and Hypertension, Graduate School of Medical and Dental Sciences, Kagoshima University, 8-35-1 Sakuragaoka, Kagoshima City, Kagoshima 890-8520 Japan

**Keywords:** Cardiac tamponade, Pericardiocentesis, Coronary artery perforation, Coil embolization

## Abstract

**Background:**

Pericardiocentesis is frequently performed when fluid needs to be removed from the pericardial sac, for both therapeutic and diagnostic purposes, however, it can still be a high-risk procedure in inexperienced hands and/or an emergent setting.

**Case presentation:**

A 78-year-old male made an emergency call complaining of the back pain. When the ambulance crew arrived at his home, he was in a state of shock due to cardiac tamponade diagnosed by portable echocardiography. The pericardiocentesis was performed using a puncture needle on site, and the patient was immediately transferred to our hospital by helicopter. Contrast-enhanced computed tomography showed a small protrusion of contrast media on the inferior wall of the left ventricle, suggesting cardiac rupture due to acute myocardial infarction. Emergency coronary angiography was then performed, which confirmed occlusion of the posterior descending branch of the left circumflex coronary artery. In addition, extravasation of contrast medium due to coronary artery perforation was observed in the acute marginal branch of the right coronary artery. We considered that coronary artery perforation had occurred as a complication of the pericardial puncture. We therefore performed transcatheter coil embolization of the perforated branch, and angiography confirmed immediate vessel sealing and hemostasis. After the procedure, the patient made steady progress without a further increase in pericardial effusion, and was discharged on the 50th day after admission.

**Conclusions:**

When performing pericardial drainage, it is important that the physician recognizes the correct procedure and complications of pericardiocentesis, and endeavors to minimize the occurrence of serious complications. As with the patient presented, coil embolization is an effective treatment for distal coronary artery perforation caused by pericardiocentesis.

## Background

Cardiac tamponade is a life-threatening condition caused by compression of the heart due to accumulation of fluid within the pericardial space. Aortic dissection, acute myocardial infarction and interventional procedures, in addition to pericardial diseases of any etiology, may cause cardiac tamponade. Pericardiocentesis is an important procedure for treating cardiac tamponade, and echocardiography-guided pericardiocentesis has been recognized as a safe and feasible technique that has low complication rates. However, it can still be a high-risk procedure in inexperienced hands and/or an emergency setting [1]. Here we report a case of right coronary artery perforation secondary to pericardiocentesis for cardiac tamponade.

## Case presentation

**Fig. 1:**
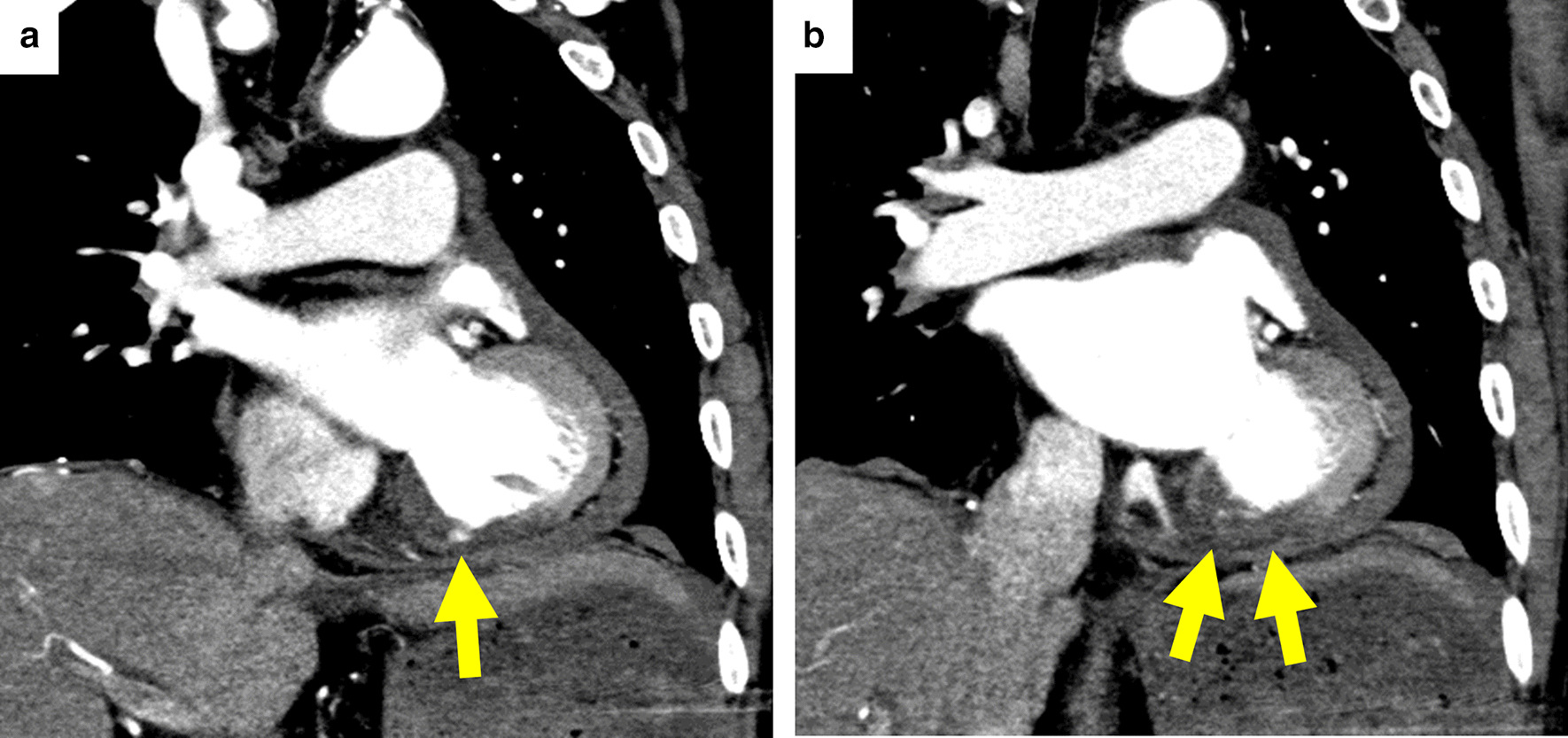
Contrast-enhanced computed tomography at the focus of left ventricle. Contrast-enhanced computed tomography shows partial protrusion of contrast medium from the inferior wall of the left ventricle (**a**) and poor enhancement of the inferior wall (**b**)

**Fig. 2:**
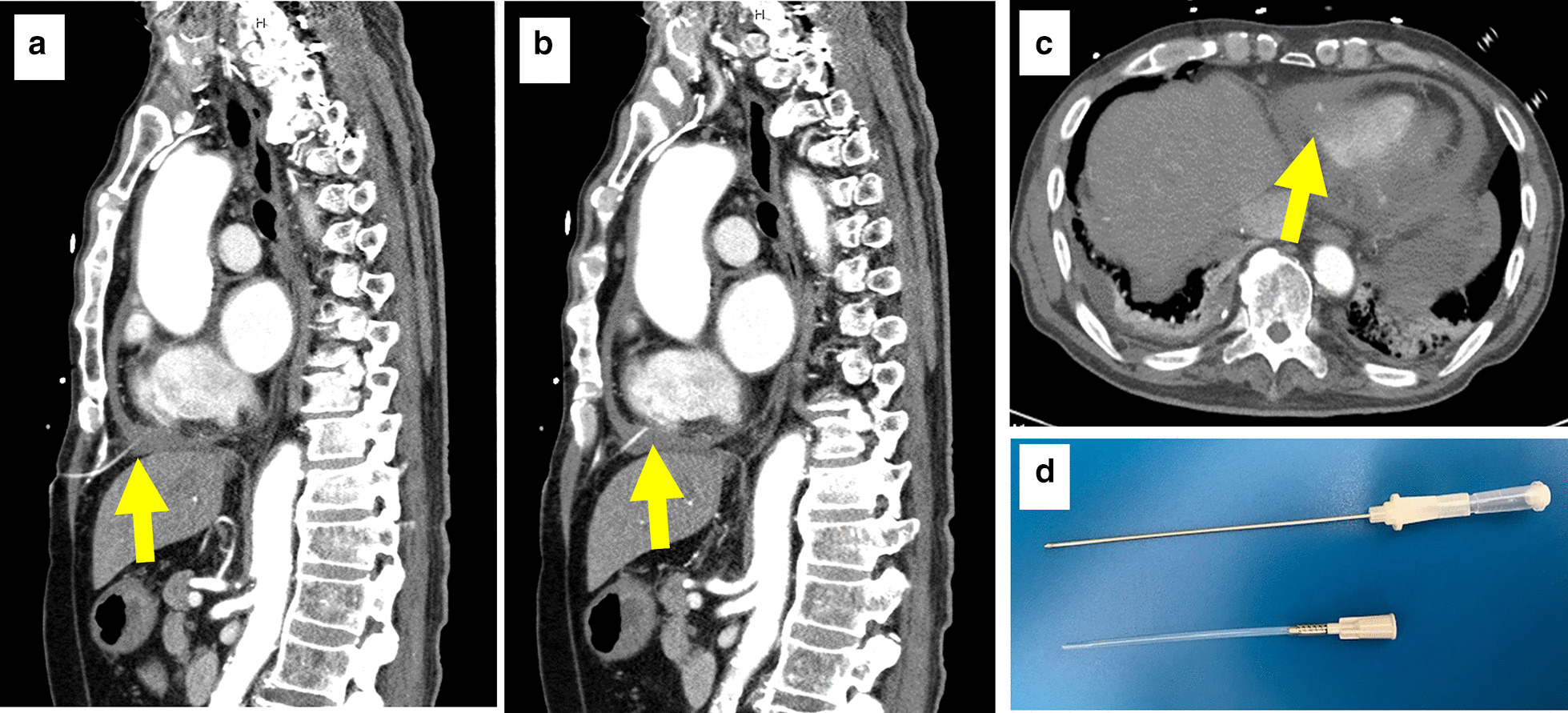
Pericardiocentesis and puncture needle**.** Computed tomography shows the tip of the outer cannula of the puncture needle in the pericardial cavity (**a**–**c**). Needle used for pericardiocentesis (**d**)

**Fig. 3:**
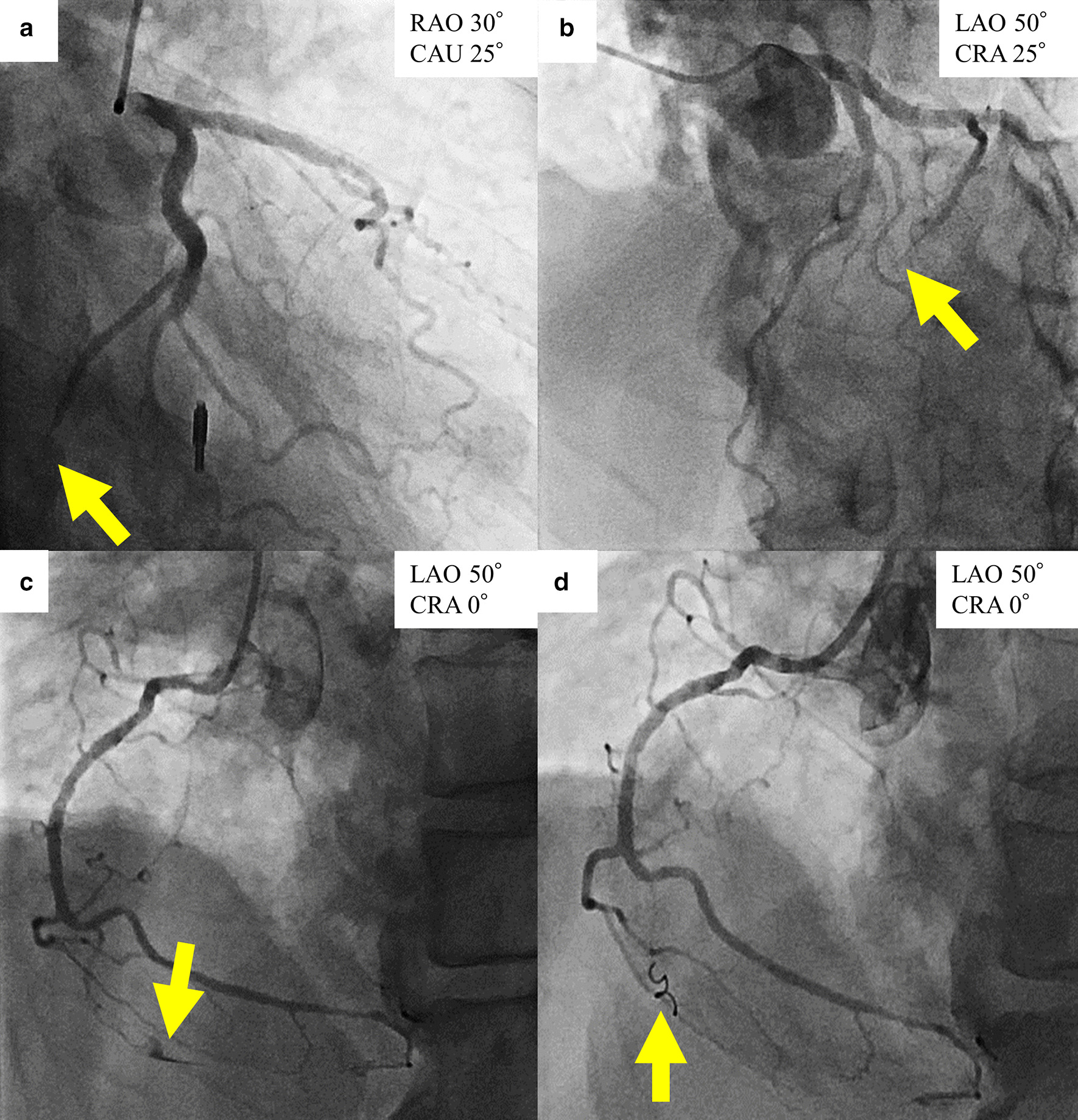
Coronary angiography and coil embolization. Left coronary angiography reveals occlusion of the posterior descending branch of the left circumflex coronary artery (**a**, **b**). Right coronary angiography shows extravasation of contrast medium in the acute marginal branch of the right coronary artery (**c**), and confirms sealing of the perforated artery following coil embolization (**d**). *CAU* caudal oblique, *CRA* cranial oblique, *LAO* left anterior oblique, *RAO* right anterior oblique

A 78-year-old male with chronic kidney disease, dementia and sequelae of cerebral hemorrhage presented to his family doctor complaining of back pain; however, there were no abnormal findings on physical examination. Three days later, the back pain worsened and an ambulance was called. When the ambulance crew (including an emergency doctor) arrived at his home, his systolic blood pressure was 50 mm Hg, and the doctor performed portable echocardiography on site. Cardiac tamponade was diagnosed, and pericardiocentesis was performed using a puncture needle via the subxiphoidal approach. After draining ~ 500 ml of bloody pericardial fluid, the patient’s blood pressure immediately increased to 124/98 mm Hg. The patient was transferred to our hospital by helicopter with the outer cannula of the puncture needle placed in the pericardial cavity, and his hemodynamic status was stable at the time of arrival. We immediately started intravenous administration of saline (200 mL/h) to prevent the decrease in blood pressure due to blood loss. Electrocardiogram revealed negative T waves with slight ST elevation in II, III, and aVF leads, and echocardiography revealed severe hypokinesis at the left ventricular inferior wall and a small pericardial effusion. Laboratory tests showed the abnormal values in white blood cell (12.5 × 10^9^/L, reference range, 3.3 to 8.6 × 10^9^/L), C-reactive protein levels (68.5 mg/L, reference range < 1.4 mg/L), creatinine (1.24 mg/dL, reference range, 0.46–0.79 mg/dL), and highly sensitive troponin I (6559 ng/L, reference range < 34.2 ng/L). Creatinine phosphokinase levels (101 IU/L, reference range, 41 to 153 IU/L), hemoglobin (12.3 g/L, reference range, 11.6 to 14.8 g/L), and platelets (254 × 10^9^/L, reference range, 158 to 348 × 10^9^/L) were within reference range. We quickly performed contrast-enhanced computed tomography of the chest. A small protrusion of contrast media was observed on the inferior wall of the left ventricle, suggesting cardiac rupture due to acute myocardial infarction (AMI) (Fig. 1). The tip of the outer cannula was confirmed in the pericardial cavity (Fig. 2) and appeared to reach epicardial adipose tissue around the right ventricle (Fig. 2b). Emergency coronary angiography was then performed, which confirmed occlusion of the posterior descending branch of the left circumflex coronary artery (Fig. 3a, b). Stenotic or occluded lesions were not found in other coronary arteries, and the patient's coronary tree was left side dominant. In addition, extravasation of contrast medium due to Ellis type III coronary artery perforation was observed in the acute marginal branch of the right coronary artery (Fig. 3c, Additional file 1: Video 1). We considered that coronary artery perforation had occurred as a complication of the pericardial puncture, and we performed transcatheter coil embolization of the perforated branch. A hydrophilic microcatheter (internal diameter, 0.018-in.; MIZUKI standard, KANEKA, Japan) was advanced selectively immediately proximal to the site of leakage in the acute marginal branch. One 0.018-in. and 20-mm-long tapered microcoil (diameter, 2 mm; Hilal, Cook, USA) was quickly released using the microcatheter, and angiography confirmed immediate vessel sealing and hemostasis 170 min after the emergency pericardiocentesis (Fig. 3d, Additional file 2: Video 2). After the coil embolization, there was no new bloody pericardial effluent and the patient was hemodynamically stable. Therefore, we reduced the rate of intravenous saline administration to 1 mL/kg/h. The total amount of drained bloody pericardial fluid was 610 ml including the first 500 ml. Hemoglobin level decreased to 8.9 g/L, and we performed blood transfusion. The total amount of contrast medium used in contrast-enhanced computed tomography and coronary angiography was 165 ml. Creatinine increased to 1.89 mg/dL 48 h after the use of contrast medium, and the urine volume decreased. Therefore, intravenous administration of saline continued, and then the urine volume gradually increased. Consequently, Intravenous administration of saline had been performed for 7 days. Timeline of clinical presentation and treatment was presented in Table 1.

**Table 1:** Timeline of clinical presentation and treatment

Time	Clinical presentation and treatment
28th February	78-year-old male presented to his family doctor complaining of back pain
2nd March, 9:40 a.m.	The back pain worsened and an ambulance was called
2nd March, 10:50 a.m.	Pericardiocentesis was performed on site
2nd March, 11:20 a.m.	The patient was transferred to our hospital by helicopter, and intravenous administration of saline was started
2nd March, 1:40 p.m.	Coil embolization was performed for coronary artery perforation
Day 1 of admission	There was no new bloody pericardial effluent
Day 2 of admission	Contrast-induced nephropathy occurred
Day 3 of admission	Antibiotics administration for aspiration pneumonia was started
Day 7 of admission	Intravenous administration of saline was stopped
Day 10 of admission	The patient was transferred from Intensive Care Unit to High Care Unit
Day 14 of admission	The patient was transferred from High Care Unit to general ward
Day 24 of admission	Antibiotics administration for aspiration pneumonia was stopped
Day 50 of admission	The patient was discharged in a stable condition

Oozing-type cardiac rupture due to AMI was considered as a cause of the cardiac tamponade, but the time of onset of AMI could not be identified, and subsequent blood tests had shown no increase in cardiac enzyme levels. Considering the general condition of the patient, who had dementia and comorbid chronic kidney disease, and the requests of the patient and his family, the patient underwent conservative treatment without cardiac surgery for oozing-type cardiac rupture and any procedure for occlusion of the posterior descending branch of the left circumflex coronary artery. Although the patient made steady progress without a further increase in pericardial effusion after the procedure, the patient needed treatment for a concomitant aspiration pneumonia (from day 3 to day 24 of admission) and rehabilitation for muscle weakness due to protracted bed rest. Finally, the patient was discharged on the 50th day after admission. Creatinine level was 1.28 mg/dL at the time of discharge.

## Discussion and conclusion

Percutaneous drainage is an important treatment strategy for evacuating a pericardial effusion. Urgent pericardiocentesis is required in each case of cardiac tamponade and hemodynamic shock, and cannot be delayed. Although urgent pericardiocentesis becomes a mandatory and life-saving procedure, there is a risk of serious complications. The most serious complications of pericardiocentesis are laceration and perforation of the myocardium and coronary arteries, which are reported in < 1% of procedures [2, 3], but may necessitate cardiac surgery. The Working Group on Myocardial and Pericardial Diseases of the European Society of Cardiology [4] has recommended the use of echocardiography in performing pericardiocentesis in order to prevent complications, with the recommendations that a soft-tip J guidewire should be promptly inserted as soon as the tip of the needle is in the pericardial space, and that a pig-tail catheter or a standard 7F central venous catheter should be inserted for drainage of the effusion.

In the present case, the emergency doctor performed pericardial puncture on site for cardiac tamponade with hemodynamic shock. Although the doctor performed pericardiocentesis using portable echocardiography, he did not have a soft-tip J guidewire, a pig-tail catheter, or a 7F venous catheter. Therefore, there was no choice but to use a puncture needle, and the outer cannula was placed in the pericardial cavity. Consequently, the pericardiocentesis induced Ellis type III coronary artery perforation, which is the most severe type of coronary perforation [5]. Al‐Lamee et al. [6] reported an in‐hospital mortality rate of 14.8% among patients with Ellis type III coronary artery perforation. Several reports have demonstrated that endovascular treatment for coronary artery perforation may be equally effective and more expedient than emergent bypass surgery in terms of sealing the leaking vessel [7, 8]. Compared with surgical procedures for the treatment of coronary perforation, coils can be deployed easily and rapidly through normal guide catheters or through microcatheters, and inserted accurately to the site of perforation [9]. We also performed coil embolization easily, and completely sealed the perforated vessel.

In this case, the pericardiocentesis performed by the emergency doctor saved the patient’s life but resulted in coronary artery perforation, which is associated with high mortality. Therefore, when performing pericardial drainage, it is important that the physician recognizes the correct procedure and complications of pericardiocentesis, and endeavors to minimize the occurrence of serious complications.

The emergency doctor worked in a secondary emergency hospital in this case, and he was not a cardiologist. Our institution is a tertiary hospital with 2 catheter rooms and 5 interventional cardiologists. We can perform more than 200 cases of primary and elective percutaneous coronary interventions per year.

To the best of our knowledge, this is a rare instance of coil embolization performed for coronary perforation caused by pericardiocentesis for cardiac tamponade. Coil embolization is an effective treatment for distal coronary artery perforation caused by pericardiocentesis.

## Supplementary Information

**Additional file 1. **Coronary artery perforation in the acute marginal branch of the right coronary artery.

**Additional file 2. **Coil embolization for coronary artery perforation. 

## Data Availability

The datasets used and/or analyzed during the current study are available from the corresponding author on reasonable request.

## Abbreviations

AMI: Acute myocardial infarction
